# Hybrid class of robust type estimators for variance estimation using mean and variance of auxiliary variable

**DOI:** 10.1016/j.heliyon.2024.e31039

**Published:** 2024-05-11

**Authors:** Mohammed Ahmed Alomair, Syed Aflake Hussain Shah Gardazi

**Affiliations:** a Department of Quantitative Methods, School of Business, King Faisal University, Al-Ahsa, 31982, Saudi Arabia; b Department of Mathematics and Statistics, Ripah International University, Islamabad, 46000, Pakistan

**Keywords:** Auxiliary information, Robust measures, Study variable, Variance estimation

## Abstract

This study is completed for the estimation of unknown population variance for the variable of mean and variance of interest. To accomplish this task, a new generalized class of robust kind of variance estimators proposed utilizing known descriptives of auxiliary variable, for example, Mid-range, Hodges-Lehmann Mean, Tri-mean, deciles mean, coefficient of skewness, interquartile range, first quartile, coefficient of kurtosis, semi-interquartile average, inter decile range and Mean, etc. These conventional measures of auxiliary variable improve the accuracy of the suggested class under simple random sampling without replacement (SRSWOR) scheme. The properties such as the bias, mean square errors (MSE), and least MSE of the suggested class are derived up to first order of approximation. The superiority conditions of the developed class of estimators over existing estimators are also made out theoretically. Finally, numerical representation is also completed for the motivations behind the article. The usual variance estimator is considered as a benchmark for comparing all considered estimators in numerical illustration. The results have been indicated that the suggested class is performing better than the usual variance estimator and all other thoughts about existing estimators.

## 1 Introduction

The utilization of auxiliary information to estimate the unknown population mean of interest to increase the efficiency of estimators in sample surveys is a widely settled practice. Much work has been done over the past few years for the estimation of population variance of the research variable when complete data is available about the research and additional variables, see Refs. [[1], [2], [3], [4]]. In this field of study, product, ratio, and regression estimation approaches are good examples.

By using supplementary data, we are able to obtain more accurate population parameter estimations, see Refs. [[5], [6], [7], [8], [9]]. It is a commonly settled practice to use additional information to increase the accuracy of estimation methods. Numerous researchers used known population parameters of auxiliary information, such as coefficient of variation, mid-range, coefficient of skewness, semi-interquartile range, coefficient of kurtosis, semi-interquartile average and inter decile range of the additional variable and the correlation coefficient between the research and additional variable to improve the accuracy of the estimation methods of population mean. Similarly [10], introduced a family of ratio-type variance estimation methods. Recently [11], focused on improved population variance estimation using robust measures. References [[12], [13], [14], [15]] developed L-moments based robust variance estimators. Taking motivation from these, we define a generalized class of robust type estimation methods for the estimation of population variance of the research variable which is more efficient than existing estimators. It is entrusting to note that the new generalized robust class of estimation methods is not only efficient in the presence of extreme values but also in the absence of extreme values.

The remaining part of the paper is defined as follows. Section 2 documented some important notations and some existing estimation methods of the population variance of the research variable. In Section 3 the generalized class of estimators has been defined and the expressions for its bias and MSE have been obtained to first order of approximation. In Section 4 we theoretically explained the efficiency conditions of proposed and existing estimators. Section 5 covers the numerical application of existing and proposed work. Finally, the article ended with concluding remarks.

## 2 Existing classes of variance estimators

Assume that there are N transitive units and in a finite population *G*. Let (Y,X) represent the research and the supplementary variables that are identified on *G*, resulting in values $\left(y_{i},x_{i}\right)$ on G(i=1,2…N) respectively. Let there be a high correlation between the population variance $\mu_{20}$ of the research data Y and the population variance $\mu_{02}$ of the supplementary data X. Let a simple random sample (SRS) of size n be chosen without replacement (WOR) sampling from the population G.

Some useful notations are for the purpose of the article as given below:

$\mu_{y}=\frac{\sum_{i}^{N}y_{i}}{N}$: the population mean of Y,
${\hat{\mu }}_{y}=\frac{\sum_{i}^{n}y_{i}}{n}$: the sample mean of Y,
$\mu_{x}=\frac{\sum_{i}^{N}x_{i}}{N}$: the population mean of X,
${\hat{\mu }}_{x}=\frac{\sum_{i}^{n}x_{i}}{n}$: the sample mean of X,
$\mu_{20}=\frac{\sum_{i}^{N}{\left({y_{i}-\mu }_{y}\right)}^{2}}{N-1}$: the population variance of Y,
${\hat{\mu }}_{20}=\frac{\sum_{i}^{n}{\left(y_{i}-{\hat{\mu }}_{y}\right)}^{2}}{n-1}$: the sample variance of Y,
$\mu_{02}=\frac{\sum_{i}^{N}{\left({x_{i}-\mu }_{x}\right)}^{2}}{N-1}$: the population variance of X,
${\hat{\mu }}_{02}=\frac{\sum_{i}^{n}{\left(x_{i}-{\hat{\mu }}_{x}\right)}^{2}}{n-1}$: the sample variance of X,
$\mu_{yx}=\frac{\sum_{i}^{N}\left({x_{i}-\mu }_{x}\right)\left({y_{i}-\mu }_{y}\right)}{N-1}\text{:}$ the population covariance of X and Y,
${\hat{\mu }}_{yx}=\frac{\sum_{i}^{n}\left(x_{i}-{\hat{\mu }}_{x}\right)\left(y_{i}-{\hat{\mu }}_{y}\right)}{n-1}$ the sample covariance of X and Y,
$\vartheta_{03}=\frac{\sum_{i}^{N}{\left({y_{i}-\mu }_{y}\right)}^{3}}{N-1}$: the third central moment of X,
$\mu_{04}=\frac{\sum_{i}^{N}{\left({x_{i}-\mu }_{x}\right)}^{4}}{N-1}$: the fourth central moment of X,
$\mu_{40}=\frac{\sum_{i}^{N}{\left({y_{i}-\mu }_{y}\right)}^{4}}{N-1}$: the fourth central moment of Y,
$\vartheta_{21}=\frac{\sum_{i}^{N}{\left({y_{i}-\mu }_{y}\right)}^{2}\left({x_{i}-\mu }_{x}\right)}{N}$: the ${\left(2,1\right)}^{\text{th}}$ central product moment,
$\vartheta_{22}=\frac{\sum_{i}^{N}{\left({y_{i}-\mu }_{y}\right)}^{2}{\left({x_{i}-\mu }_{x}\right)}^{2}}{N}$: the ${\left(2,2\right)}^{\text{th}}$ central product moment,
$C_{y}=\frac{\sqrt{\mu_{20}}}{\mu_{y}}$: the coefficient of variation of Y,
$C_{x}=\frac{\sqrt{\mu_{02}}}{\mu_{x}}$: the coefficient of variation of X,
ρ: Population correlation coefficient,
$M_{d}$: Median of X,
$Q_{1}$: First quartile,
$Q_{3}$: Third quartile,
$Q_{rx}=Q_{3}-Q_{1}$: Inter Quartile range,
$Q_{dx}$: Semi quartile range,
$Q_{dx}=\frac{Q_{3}+Q_{1}\;}{2}$: Inter quartile range,
$D_{ix}$: Decile (i=1,2,…,10) of X,
$M_{Tri}$: Tri-mean of X,
$M_{Rx}$: Mid-range of X,
$H_{Lx}$: Hodges-Lehmann Mean of X,
$D_{Mx}$: Deciles mean of X,
$\eta_{21}=\frac{\vartheta_{21}}{\left(\mu_{20}\mu_{02}^{1/2}\right)},{\;\eta }_{03},\frac{\vartheta_{03}^{2}}{\mu_{02}^{3}},f=\frac{n}{N},\theta_{a}=\frac{\vartheta_{22}}{\left(\mu_{20}\mu_{02}\right)},\theta_{a}^{*}=\theta_{a}-1$,
$\eta_{04}^{*}=\eta_{04}-1,\eta_{40}^{*}=\eta_{40}-1,{\;\eta }_{40}=\frac{\mu_{40}}{\mu_{20}},\eta_{04}=\frac{\mu_{04}}{\mu_{04}}$ with $\vartheta_{rs}=\frac{\sum_{i}^{N}{\left({y_{i}-\mu }_{y}\right)}^{r}{\left({x_{i}-\mu }_{x}\right)}^{s}}{N}$, (r,s) being non-negative integer;
$\delta_{0}=\frac{\left({\hat{\mu }}_{20}-\mu_{20}\right)}{\mu_{20}},\delta_{1}=\frac{\left({\hat{\mu }}_{02}-\mu_{02}\right)}{\mu_{02}},\delta_{2}=\frac{\left({\hat{\mu }}_{x}-\mu_{x}\right)}{\mu_{x}}$.

The usual unbiased estimator of $\mu_{20}$ is

(1) $${\hat{g}}_{0}={\hat{\mu }}_{20}=\frac{\sum_{i}^{n}{\left(y_{i}-{\hat{\mu }}_{y}\right)}^{2}}{n-1}$$

In equation (1), ${\hat{\mu }}_{y}$ is the sample mean of Y.

Thus, the asymptomatic variance of ${\hat{g}}_{0}$ is

(2) $$V\left({\hat{g}}_{0}\right)=b\mu_{40}\eta_{04\;}^{*}$$

In equation (2), $b=n^{-1}$.

Remarks: For simplification, it is noted from the literature that (fpc) is ignored.

Recently [11], have introduced the following ratio type class-I estimators, using known additional $\mu_{02}$ given as under

(3) $$T_{Aj}=L_{A}{\hat{\mu }}_{20}\left(\frac{\alpha \mu_{x}+b}{\alpha {\hat{\mu }}_{x}+b}\right)$$

In equation (3), $L_{A}$ is derived constant and (a,b) be known auxiliary information of X.

$$E\left(\delta_{0}^{2}\right)=b\eta_{04}^{*},E\left(\delta_{2}^{2}\right)=bC_{x}^{2},E\left(\delta_{0}\delta_{2}\right)=b\eta_{21}C_{x}$$

The bias and asymptotic MSE is shown in equation (4) and equation (5) as:

(4) $$B\left(T_{Aj}\right)=\mu_{20}\left\{\left(L_{A}-1\right)+bL_{A}\left(\eta_{j_{2}^{\prime \prime }}C_{x}^{2}-\eta_{j}^{\prime \prime }\eta_{21}C_{x}\right)\right\}$$

and

(5) $$MSE{\left(T_{Aj}\right)}_{\min}=\mu_{40}\left(\frac{M_{a}^{2}}{M_{b}}\right)$$

where

$$\eta_{j}^{\prime \prime }=\frac{a\mu_{x}}{a\mu_{x}+b}{,M}_{a}=1+bC_{x}\eta_{j}^{\prime \prime }\left(C_{x}\eta_{j}^{\prime \prime }-\eta_{21}\right),{\;M}_{b}=1+b\left\{\eta_{40}^{*}+C_{c}\eta_{j}^{\prime \prime }\left(3C_{x}\eta_{j}^{\prime \prime }-4\eta_{21}\right)\right\}\;\text{.}$$

Some Family members of $T_{Aj}$ are displayed in Table 1.

**Table 1:** Family members of $T_{Aj}$.

Estimator	Values of constant	
	a	b
$T_{A1}=L_{A}{\hat{\mu }}_{20}\left(\frac{{\;\eta }_{03}\mu_{x}+M_{Tri}}{{\;\eta }_{03}{\hat{\mu }}_{x}+M_{Tri}}\right)$	${\;\eta }_{03}$	$M_{Tri}$
$T_{A2}=L_{A}{\hat{\mu }}_{20}\left(\frac{{\;\eta }_{03}\mu_{x}+M_{Rx}}{{\;\eta }_{03}{\hat{\mu }}_{x}+M_{Rx}}\right)$	${\;\eta }_{03}$	$M_{Rx}$
$T_{A3}=L_{A}{\hat{\mu }}_{20}\left(\frac{{\;\eta }_{03}\mu_{x}+H_{Lx}}{{\;\eta }_{03}{\hat{\mu }}_{x}+H_{Lx}}\right)$	${\;\eta }_{03}$	$H_{Lx}$
$T_{A4}=L_{A}{\hat{\mu }}_{20}\left(\frac{{\;\eta }_{03}\mu_{x}+D_{Mx}}{{\;\eta }_{03}{\hat{\mu }}_{x}+D_{Mx}}\right)$	${\;\eta }_{03}$	$D_{Mx}$
$T_{A5}=L_{A}{\hat{\mu }}_{20}\left(\frac{C_{x}\mu_{x}+M_{Tri}}{C_{x}{\hat{\mu }}_{x}+M_{Tri}}\right)$	$C_{x}$	$M_{Tri}$
$T_{A6}=L_{A}{\hat{\mu }}_{20}\left(\frac{C_{x}\mu_{x}+M_{Rx}}{C_{x}{\hat{\mu }}_{x}+M_{Rx}}\right)$	$C_{x}$	$M_{Rx}$
$T_{A7}=L_{A}{\hat{\mu }}_{20}\left(\frac{C_{x}\mu_{x}+H_{Lx}}{C_{x}{\hat{\mu }}_{x}+H_{Lx}}\right)$	$C_{x}$	$H_{Lx}$
$T_{A8}=L_{A}{\hat{\mu }}_{20}\left(\frac{C_{x}\mu_{x}+D_{Mx}}{C_{x}{\hat{\mu }}_{x}+D_{Mx}}\right)$	$C_{x}$	$D_{Mx}$
$T_{A9}=L_{A}{\hat{\mu }}_{20}\left(\frac{\rho \mu_{x}+M_{Tri}}{\rho {\hat{\mu }}_{x}+M_{Tri}}\right)$	ρ	$M_{Tri}$
$T_{A10}=L_{A}{\hat{\mu }}_{20}\left(\frac{\rho \mu_{x}+M_{Rx}}{\rho {\hat{\mu }}_{x}+M_{Rx}}\right)$	ρ	$M_{Rx}$
$T_{A11}=L_{A}{\hat{\mu }}_{20}\left(\frac{\rho \mu_{x}+H_{Lx}}{\rho {\hat{\mu }}_{x}+H_{Lx}}\right)$	ρ	$H_{Lx}$
$T_{A12}=L_{A}{\hat{\mu }}_{20}\left(\frac{\rho \mu_{x}+D_{Mx}}{\rho {\hat{\mu }}_{x}+D_{Mx}}\right)$	ρ	$D_{Mx}$
$T_{A13}=L_{A}{\hat{\mu }}_{20}\left(\frac{{\;\eta }_{04}\mu_{x}+M_{Tri}}{{\;\eta }_{04}{\hat{\mu }}_{x}+M_{Tri}}\right)$	${\;\eta }_{04}$	$M_{Tri}$
$T_{A14}=L_{A}{\hat{\mu }}_{20}\left(\frac{{\;\eta }_{04}\mu_{x}+M_{Rx}}{{\;\eta }_{04}{\hat{\mu }}_{x}+M_{Rx}}\right)$	${\;\eta }_{04}$	$M_{Rx}$
$T_{A15}=L_{A}{\hat{\mu }}_{20}\left(\frac{{\;\eta }_{04}\mu_{x}+H_{Lx}}{{\;\eta }_{04}{\hat{\mu }}_{x}+H_{Lx}}\right)$	${\;\eta }_{04}$	$H_{Lx}$
$T_{A16}=L_{A}{\hat{\mu }}_{20}\left(\frac{{\;\eta }_{04}\mu_{x}+D_{Mx}}{{\;\eta }_{04}{\hat{\mu }}_{x}+D_{Mx}}\right)$	${\;\eta }_{04}$	$D_{Mx}$

Further [11], have also introduced the following ratio type Class-II estimators, using known additional $\mu_{02}$ is given in equation (6), as

(6) $$T_{Bj}=L_{B}{\hat{\mu }}_{20}{\left(\frac{a\mu_{x}+b}{a{\hat{\mu }}_{x}+b}\right)}^{\frac{C\mu_{x}}{C\mu_{x}+d}}$$

where, $L_{B}$ is derived constant and (a,b,c,d) being either auxiliary variables.

The bias and the asymptotic MSE are shown in equation (7) and equation (8) as:

(7) $$B\left(T_{Bj}\right)=\mu_{20}\left[\left(L_{B}-1\right)+bL_{B}{\hat{\eta }}_{a}C_{x}\left\{\frac{{\hat{\eta }}_{b}^{2}+{\hat{\eta }}_{b}}{2}{\hat{\eta }}_{b}C_{x}-{\hat{\eta }}_{b}\eta_{21}\right\}\right]$$

and

(8) $$MSE{\left(T_{Bj}\right)}_{\min}=\mu_{40}\left(1-\frac{M_{c}^{2}}{M_{d}}\right)$$

where

$${\hat{\eta }}_{a}=\frac{a\mu_{x}}{a\mu_{x}+b},{\hat{\eta }}_{b}=\frac{C\mu_{x}}{C\mu_{x}+d},M_{c}=1+\frac{b}{2}C_{x}{\hat{\eta }}_{a}\left\{\left({\hat{\eta }}_{b}^{2}+{\hat{\eta }}_{b}\right){\hat{\eta }}_{a}C_{x}-2{\hat{\eta }}_{b}\eta_{21}\right\}\text{,}$$

$$M_{d}=1+b\left\{\eta_{40}^{*}+\left(2{\hat{\eta }}_{b}^{2}+{\hat{\eta }}_{b}\right){\hat{\eta }}_{a}^{2}C_{x}^{2}-4{\hat{\eta }}_{a}{\hat{\eta }}_{b}\eta_{21}C_{x}\right\}\;\text{.}$$

Some family members of $T_{Bj}$ are displayed in Table 2.

**Table 2:** Family members of $T_{Bj}$.

Estimator	Values of constant			
	a	b	C	d
$T_{B1}=L_{B}{\hat{\mu }}_{20}{\left(\frac{{\;\eta }_{03}\mu_{x}+M_{Tri}}{{\;\eta }_{03}{\hat{\mu }}_{x}+M_{Tri}}\right)}^{\frac{{\;\eta }_{03}\mu_{x}}{{\;\eta }_{03}\mu_{x}+M_{Tri}}}$	${\;\eta }_{03}$	$M_{Tri}$	${\;\eta }_{03}$	$M_{Tri}$
$T_{B2}=L_{B}{\hat{\mu }}_{20}{\left(\frac{{\;\eta }_{03}\mu_{x}+M_{Rx}}{{\;\eta }_{03}{\hat{\mu }}_{x}+M_{Rx}}\right)}^{\frac{{\;\eta }_{03}\mu_{x}}{{\;\eta }_{03}\mu_{x}+M_{Rx}}}$	${\;\eta }_{03}$	$M_{Rx}$	${\;\eta }_{03}$	$M_{Rx}$
$T_{B3}=L_{B}{\hat{\mu }}_{20}{\left(\frac{{\;\eta }_{03}\mu_{x}+H_{Lx}}{{\;\eta }_{03}{\hat{\mu }}_{x}+H_{Lx}}\right)}^{\frac{{\;\eta }_{03}\mu_{x}}{{\;\eta }_{03}\mu_{x}+H_{Lx}}}$	${\;\eta }_{03}$	$H_{Lx}$	${\;\eta }_{03}$	$H_{Lx}$
$T_{B4}=L_{B}{\hat{\mu }}_{20}{\left(\frac{{\;\eta }_{03}\mu_{x}+D_{Mx}}{{\;\eta }_{03}{\hat{\mu }}_{x}+D_{Mx}}\right)}^{\frac{{\;\eta }_{03}\mu_{x}}{{\;\eta }_{03}\mu_{x}+D_{Mx}}}$	${\;\eta }_{03}$	$D_{Mx}$	${\;\eta }_{03}$	$D_{Mx}$
$T_{B5}=L_{B}{\hat{\mu }}_{20}{\left(\frac{C_{x}\mu_{x}+M_{Tri}}{C_{x}{\hat{\mu }}_{x}+M_{Tri}}\right)}^{\frac{C_{x}\mu_{x}}{C_{x}\mu_{x}+M_{Tri}}}$	$C_{x}$	$M_{Tri}$	$C_{x}$	$M_{Tri}$
$T_{B6}=L_{B}{\hat{\mu }}_{20}{\left(\frac{C_{x}\mu_{x}+M_{Rx}}{C_{x}{\hat{\mu }}_{x}+M_{Rx}}\right)}^{\frac{C_{x}\mu_{x}}{C_{x}\mu_{x}+M_{Rx}}}$	$C_{x}$	$M_{Rx}$	$C_{x}$	$M_{Rx}$
$T_{B7}=L_{B}{\hat{\mu }}_{20}{\left(\frac{C_{x}\mu_{x}+H_{Lx}}{C_{x}{\hat{\mu }}_{x}+H_{Lx}}\right)}^{\frac{C_{x}\mu_{x}}{C_{x}\mu_{x}+H_{Lx}}}$	$C_{x}$	$H_{Lx}$	$C_{x}$	$H_{Lx}$
$T_{B8}=L_{B}{\hat{\mu }}_{20}{\left(\frac{C_{x}\mu_{x}+D_{Mx}}{C_{x}{\hat{\mu }}_{x}+D_{Mx}}\right)}^{\frac{C_{x}\mu_{x}}{C_{x}\mu_{x}+D_{Mx}}}$	$C_{x}$	$D_{Mx}$	$C_{x}$	$D_{Mx}$
$T_{B9}=L_{B}{\hat{\mu }}_{20}{\left(\frac{\rho \mu_{x}+M_{Tri}}{\rho {\hat{\mu }}_{x}+M_{Tri}}\right)}^{\frac{\rho \mu_{x}}{\rho \mu_{x}+M_{Tri}}}$	ρ	$M_{Tri}$	ρ	$M_{Tri}$
$T_{B10}=L_{B}{\hat{\mu }}_{20}{\left(\frac{\rho \mu_{x}+M_{Rx}}{\rho {\hat{\mu }}_{x}+M_{Rx}}\right)}^{\frac{\rho \mu_{x}}{\rho \mu_{x}+M_{Rx}}}$	ρ	$M_{Rx}$	ρ	$M_{Rx}$
$T_{B11}=L_{B}{\hat{\mu }}_{20}{\left(\frac{\rho \mu_{x}+H_{Lx}}{\rho {\hat{\mu }}_{x}+H_{Lx}}\right)}^{\frac{\rho \mu_{x}}{\rho \mu_{x}+H_{Lx}}}$	ρ	$H_{Lx}$	ρ	$H_{Lx}$
$T_{B12}=L_{B}{\hat{\mu }}_{20}{\left(\frac{\rho \mu_{x}+D_{Mx}}{\rho {\hat{\mu }}_{x}+D_{Mx}}\right)}^{\frac{\rho \mu_{x}}{\rho \mu_{x}+D_{Mx}}}$	ρ	$D_{Mx}$	ρ	$D_{Mx}$
$T_{B13}=L_{B}{\hat{\mu }}_{20}{\left(\frac{{\;\eta }_{04}\mu_{x}+M_{Tri}}{{\;\eta }_{04}{\hat{\mu }}_{x}+M_{Tri}}\right)}^{\frac{{\;\eta }_{04}\mu_{x}}{{\;\eta }_{04}\mu_{x}+M_{Tri}}}$	${\;\eta }_{04}$	$M_{Tri}$	${\;\eta }_{04}$	$M_{Tri}$
$T_{B14}=L_{B}{\hat{\mu }}_{20}{\left(\frac{{\;\eta }_{04}\mu_{x}+M_{Rx}}{{\;\eta }_{04}{\hat{\mu }}_{x}+M_{Rx}}\right)}^{\frac{{\;\eta }_{04}\mu_{x}}{{\;\eta }_{04}\mu_{x}+M_{Rx}}}$	${\;\eta }_{04}$	$M_{Rx}$	${\;\eta }_{04}$	$M_{Rx}$
$T_{B15}=L_{B}{\hat{\mu }}_{20}{\left(\frac{{\;\eta }_{04}\mu_{x}+H_{Lx}}{{\;\eta }_{04}{\hat{\mu }}_{x}+H_{Lx}}\right)}^{\frac{{\;\eta }_{04}\mu_{x}}{{\;\eta }_{04}\mu_{x}+H_{Lx}}}$	${\;\eta }_{04}$	$H_{Lx}$	${\;\eta }_{04}$	$H_{Lx}$
$T_{B16}=L_{B}{\hat{\mu }}_{20}{\left(\frac{{\;\eta }_{04}\mu_{x}+D_{Mx}}{{\;\eta }_{04}{\hat{\mu }}_{x}+D_{Mx}}\right)}^{\frac{{\;\eta }_{04}\mu_{x}}{{\;\eta }_{04}\mu_{x}+D_{Mx}}}$	${\;\eta }_{04}$	$D_{Mx}$	${\;\eta }_{04}$	$D_{Mx}$

Furthermore [11], have also introduced the following ratio type Class-III estimators, using known auxiliary $\mu_{02}$ given in equation (9), as under,

(9) $$T_{Cj}=L_{C}\;\left[{\hat{\mu }}_{20}{\left(\frac{{g_{1}\mu }_{x}+g_{2}}{g_{1}{\hat{\mu }}_{x}+g_{2}}\right)}^{\frac{g_{3\mu_{x}}}{g_{3}\mu_{x}+g_{4}}}+K_{C}\;\left(\mu_{x}\;-\;{\hat{\mu }}_{x}\right)\;\right]$$

where, $L_{C}$ is derived constants and $\left(g_{1},g_{2},g_{3},g_{4}\right)$ being either auxiliary variables, whereas $K_{C}$ is the regression coefficient between the study and auxiliary variables.

The bias and the asymptotic MSE are shown in equation (10) and equation (11) as:

(10) $$B\left(T_{Cj}\right)=\mu_{20}\left[\left(L_{c}-1\right)+bL_{C}{\hat{\eta }}_{1}C_{x}\left\{\frac{{\hat{\eta }}_{2}^{2}+{\hat{\eta }}_{2}}{2}{\hat{\eta }}_{1}C_{x}-{\hat{\eta }}_{2}\eta_{21}\right\}\right]$$

and

(11) $$MSE{\left(T_{Cj}\right)}_{\min}=\left(\mu_{40}-\frac{M_{e}^{2}}{M_{f}}\right)$$

where

$${\hat{\eta }}_{1}=\frac{g_{1}\mu_{x}}{g_{1}\mu_{x}+g_{2}},{\hat{\eta }}_{2}=\frac{g_{3}\mu_{x}}{g_{3}\mu_{x}+g_{4}},M_{c}=\mu_{40}[1+\frac{b}{2}C_{x}{\hat{\eta }}_{1}\left\{\left({\hat{\eta }}_{2}^{2}+{\hat{\eta }}_{2}\right){\hat{\eta }}_{1}C_{x}-2{\hat{\eta }}_{2}\eta_{21}\right\}\text{,}$$

$$M_{f}=\mu_{40}+b\mu_{40}\left\{\eta_{40}^{*}+\left(2{\hat{\eta }}_{2}^{2}+{\hat{\eta }}_{2}\right){\hat{\eta }}_{1}^{2}C_{x}^{2}-4{\hat{\eta }}_{1}{\hat{\eta }}_{2}\eta_{21}C_{x}\right\},-2bK_{c}\mu_{20}\mu_{x}\left(\eta_{21}C_{x}-{\hat{\eta }}_{1}{\hat{\eta }}_{2}C_{x}^{2}\right)+bK_{c}{\mu_{x}^{2}C}_{x}^{2}\text{.}$$

Some family members of $T_{Cj}$ are displayed in Table 3.

**Table 3:** Family members of $T_{Cj}$.

Estimator	Values of constant			
	a	b	c	d
$T_{C1}=L_{C}{\hat{\mu }}_{20}{\left(\frac{{\;\eta }_{03}\mu_{x}+M_{Tri}}{{\;\eta }_{03}{\hat{\mu }}_{x}+M_{Tri}}\right)}^{\frac{{\;\eta }_{03}\mu_{x}}{{\;\eta }_{03}\mu_{x}+M_{Tri}}}+K_{C}\left(\mu_{x}-{\hat{\mu }}_{x}\right)$	${\;\eta }_{03}$	$M_{Tri}$	${\;\eta }_{03}$	$M_{Tri}$
$T_{C2}=L_{C}{\hat{\mu }}_{20}{\left(\frac{{\;\eta }_{03}\mu_{x}+M_{Rx}}{{\;\eta }_{03}{\hat{\mu }}_{x}+M_{Rx}}\right)}^{\frac{{\;\eta }_{03}\mu_{x}}{{\;\eta }_{03}\mu_{x}+M_{Rx}}}+K_{C}\left(\mu_{x}-{\hat{\mu }}_{x}\right)$	${\;\eta }_{03}$	$M_{Rx}$	${\;\eta }_{03}$	$M_{Rx}$
$T_{C3}=L_{C}{\hat{\mu }}_{20}{\left(\frac{{\;\eta }_{03}\mu_{x}+H_{Lx}}{{\;\eta }_{03}{\hat{\mu }}_{x}+H_{Lx}}\right)}^{\frac{{\;\eta }_{03}\mu_{x}}{{\;\eta }_{03}\mu_{x}+H_{Lx}}}+K_{C}\left(\mu_{x}-{\hat{\mu }}_{x}\right)$	${\;\eta }_{03}$	$H_{Lx}$	${\;\eta }_{03}$	$H_{Lx}$
$T_{C4}=L_{C}{\hat{\mu }}_{20}{\left(\frac{{\;\eta }_{03}\mu_{x}+D_{Mx}}{{\;\eta }_{03}{\hat{\mu }}_{x}+D_{Mx}}\right)}^{\frac{{\;\eta }_{03}\mu_{x}}{{\;\eta }_{03}\mu_{x}+D_{Mx}}}+K_{C}\left(\mu_{x}-{\hat{\mu }}_{x}\right)$	${\;\eta }_{03}$	$D_{Mx}$	${\;\eta }_{03}$	$D_{Mx}$
$T_{C5}=L_{C}{\hat{\mu }}_{20}{\left(\frac{C_{x}\mu_{x}+M_{Tri}}{C_{x}{\hat{\mu }}_{x}+M_{Tri}}\right)}^{\frac{C_{x}\mu_{x}}{C_{x}\mu_{x}+M_{Tri}}}+K_{C}\left(\mu_{x}-{\hat{\mu }}_{x}\right)$	$C_{x}$	$M_{Tri}$	$C_{x}$	$M_{Tri}$
$T_{C6}=L_{C}{\hat{\mu }}_{20}{\left(\frac{C_{x}\mu_{x}+M_{Rx}}{C_{x}{\hat{\mu }}_{x}+M_{Rx}}\right)}^{\frac{C_{x}\mu_{x}}{C_{x}\mu_{x}+M_{Rx}}}+K_{C}\left(\mu_{x}-{\hat{\mu }}_{x}\right)$	$C_{x}$	$M_{Rx}$	$C_{x}$	$M_{Rx}$
$T_{C7}=L_{C}{\hat{\mu }}_{20}{\left(\frac{C_{x}\mu_{x}+H_{Lx}}{C_{x}{\hat{\mu }}_{x}+H_{Lx}}\right)}^{\frac{C_{x}\mu_{x}}{C_{x}\mu_{x}+H_{Lx}}}+K_{C}\left(\mu_{x}-{\hat{\mu }}_{x}\right)$	$C_{x}$	$H_{Lx}$	$C_{x}$	$H_{Lx}$
$T_{C8}=L_{C}{\hat{\mu }}_{20}{\left(\frac{C_{x}\mu_{x}+D_{Mx}}{C_{x}{\hat{\mu }}_{x}+D_{Mx}}\right)}^{\frac{C_{x}\mu_{x}}{C_{x}\mu_{x}+D_{Mx}}}+K_{C}\left(\mu_{x}-{\hat{\mu }}_{x}\right)$	$C_{x}$	$D_{Mx}$	$C_{x}$	$D_{Mx}$
$T_{C9}=L_{C}{\hat{\mu }}_{20}{\left(\frac{\rho \mu_{x}+M_{Tri}}{\rho {\hat{\mu }}_{x}+M_{Tri}}\right)}^{\frac{\rho \mu_{x}}{\rho \mu_{x}+M_{Tri}}}+K_{C}\left(\mu_{x}-{\hat{\mu }}_{x}\right)$	ρ	$M_{Tri}$	ρ	$M_{Tri}$
$T_{C10}=L_{C}{\hat{\mu }}_{20}{\left(\frac{\rho \mu_{x}+M_{Rx}}{\rho {\hat{\mu }}_{x}+M_{Rx}}\right)}^{\frac{\rho \mu_{x}}{\rho \mu_{x}+M_{Rx}}}+K_{C}\left(\mu_{x}-{\hat{\mu }}_{x}\right)$	ρ	$M_{Rx}$	ρ	$M_{Rx}$
$T_{C11}=L_{C}{\hat{\mu }}_{20}{\left(\frac{\rho \mu_{x}+H_{Lx}}{\rho {\hat{\mu }}_{x}+H_{Lx}}\right)}^{\frac{\rho \mu_{x}}{\rho \mu_{x}+H_{Lx}}}+K_{C}\left(\mu_{x}-{\hat{\mu }}_{x}\right)$	ρ	$H_{Lx}$	ρ	$H_{Lx}$
$T_{C12}=L_{C}{\hat{\mu }}_{20}{\left(\frac{\rho \mu_{x}+D_{Mx}}{\rho {\hat{\mu }}_{x}+D_{Mx}}\right)}^{\frac{\rho \mu_{x}}{\rho \mu_{x}+D_{Mx}}}+K_{C}\left(\mu_{x}-{\hat{\mu }}_{x}\right)$	ρ	$D_{Mx}$	ρ	$D_{Mx}$
$T_{C13}=L_{C}{\hat{\mu }}_{20}{\left(\frac{{\;\eta }_{04}\mu_{x}+M_{Tri}}{{\;\eta }_{04}{\hat{\mu }}_{x}+M_{Tri}}\right)}^{\frac{{\;\eta }_{04}\mu_{x}}{{\;\eta }_{04}\mu_{x}+M_{Tri}}}+K_{C}\left(\mu_{x}-{\hat{\mu }}_{x}\right)$	${\;\eta }_{04}$	$M_{Tri}$	${\;\eta }_{04}$	$M_{Tri}$
$T_{C14}=L_{C}{\hat{\mu }}_{20}{\left(\frac{{\;\eta }_{04}\mu_{x}+M_{Rx}}{{\;\eta }_{04}{\hat{\mu }}_{x}+M_{Rx}}\right)}^{\frac{{\;\eta }_{04}\mu_{x}}{{\;\eta }_{04}\mu_{x}+M_{Rx}}}+K_{C}\left(\mu_{x}-{\hat{\mu }}_{x}\right)$	${\;\eta }_{04}$	$M_{Rx}$	${\;\eta }_{04}$	$M_{Rx}$
$T_{C15}=L_{C}{\hat{\mu }}_{20}{\left(\frac{{\;\eta }_{04}\mu_{x}+H_{Lx}}{{\;\eta }_{04}{\hat{\mu }}_{x}+H_{Lx}}\right)}^{\frac{{\;\eta }_{04}\mu_{x}}{{\;\eta }_{04}\mu_{x}+H_{Lx}}}+K_{C}\left(\mu_{x}-{\hat{\mu }}_{x}\right)$	${\;\eta }_{04}$	$H_{Lx}$	${\;\eta }_{04}$	$H_{Lx}$
$T_{C16}=L_{C}{\hat{\mu }}_{20}{\left(\frac{{\;\eta }_{04}\mu_{x}+D_{Mx}}{{\;\eta }_{04}{\hat{\mu }}_{x}+D_{Mx}}\right)}^{\frac{{\;\eta }_{04}\mu_{x}}{{\;\eta }_{04}\mu_{x}+D_{Mx}}}+K_{C}\left(\mu_{x}-{\hat{\mu }}_{x}\right)$	${\;\eta }_{04}$	$D_{Mx}$	${\;\eta }_{04}$	$D_{Mx}$

## 3 Hybrid class of robust type variance estimators using known auxiliary mean and variance

Using auxiliary information, taking inspiration from Ref. [11], we define the following hybrid class of estimators for unknown population variance $\mu_{20}$ of study variable Y in equation (12), as:

(12) $$F_{g}^{\prime }=\left[\omega_{1}{\hat{\mu }}_{20}\left\{\frac{\mu_{02}}{k_{1}{\hat{\mu }}_{02}+\left(1-k_{1}\mu_{02}\right)}\right\}+\omega_{2}{\hat{\mu }}_{20}{\left(\frac{\mu_{02}}{{\hat{\mu }}_{02}}\right)}^{k_{2}}\right]\;{\left(\frac{m_{1}\mu_{x}+m_{2}}{m_{1}{\hat{\mu }}_{x}+m_{2}}\right)}^{k_{3}}$$

where, *(*$m_{1},m_{2})$ are functions of the known supplementary data X such as $\mu_{x}$ *,* $C_{x}$, coefficient of kurtosis $\eta_{04}$, and ρ of the population, *(*$k_{1},k_{2},k_{3})$ being either real constant which takes values (−1,1,1),(−1,0,1), or (1,−1,−1) for designing the different estimation methods, and $\left(\omega_{1},\omega_{2}\right)$ are derived constants.

$$E\left(\delta_{2}^{2}\right)=bC_{x}^{2},E\left(\delta_{0}\delta_{2}\right)=b\eta_{21}C_{x}\;\text{and}\;\left(\delta_{1}\delta_{2}\right)=b\sqrt{\eta_{03}}C_{x}\text{.}$$

Expanding suggested family of biased estimator $F_{g}^{\prime }$ in term of $\delta^{\prime }s$, we have:

(13) $$F_{g}^{\prime }=\left\{\omega_{1}{\hat{\mu }}_{20}{\left(1+k_{1}\delta_{1}\right)}^{-1}+\omega_{2}{\hat{\mu }}_{20}{\left(1+\delta_{1}\right)}^{{-k}_{2}}\right\}{\left(1+v\delta_{2}\right)}^{{-k}_{3}}$$

Form equation (13)

(14) $$F_{g}^{\prime }-\mu_{20}=\mu_{20}\left\{\left(\omega_{1}+\omega_{2}-1\right)+\delta_{0}\left(\omega_{1}+\omega_{2}\right)-\delta_{1}\left(k_{2}\omega_{2}+{k_{1}\omega }_{1}\right)-\delta_{2}\left(k_{3}v\omega_{1}+{k_{3}v\omega }_{2}\right)\right\}-\mu_{20}\delta_{0}\delta_{1}\left(k_{2}\omega_{2}+{k_{1}\omega }_{1}\right)-\mu_{20}\delta_{0}\delta_{2}\left(k_{3}v\omega_{1}+{k_{3}v\omega }_{2}\right)-\mu_{20}\delta_{1}\delta_{2}\left(k_{2}k_{3}v\omega_{2}+k_{1}{k_{3}v\omega }_{1}\right)\;+\mu_{20}\delta_{1}^{2}\left\{\frac{K_{2}^{2}\omega_{2}+{k_{2}\omega }_{2}+2K_{1}^{2}\omega_{1}}{2}\right\}+\left\{\frac{\left(K_{3}+1\right)\left(\omega_{1}+\omega_{2}\right)}{2}\right\}k_{3}{v^{2}\delta }_{2}^{2}\;$$

Using equation (14), we can write the bias, MSE, and MSE_min_ of $F_{g}^{\prime }$ in equation (15), equation (16), and equation (17), as:

(15) $$\begin{array}{c} B\left(F_{g}^{\prime }\right)=b\mu_{20}\left\{\left(\omega_{1}+\omega_{2}-1\right)-\theta_{a}^{*}\left(k_{2}\omega_{2}+{k_{1}\omega }_{1}\right)\right\}\\ \;-b\mu_{20}\left\{\eta_{21}C_{x}\left(k_{3}v\omega_{1}+{k_{3}v\omega }_{2}\right)+\sqrt{\eta_{03}}C_{x}\left({k_{2}k}_{3}v\omega_{2}+{k_{1}k_{3}v\omega }_{1}\right)\right\} \end{array}+b\mu_{20}\left\{\eta_{04}^{*}\left\{\frac{K_{2}^{2}+\omega_{2}+2k^{2}{k_{1}\omega }_{1}}{2}\right\}+C_{x}^{2}\left\{\frac{\left(K_{3}+1\right)\left(\omega_{1}+\omega_{2}\right)}{2}K_{3}v^{2}\right\}\right\}\;$$

and

(16) $$MSE\left(F_{g}^{\prime }\right)=\mu_{40}\left(1+\omega_{1}^{2}E_{11}^{\prime }+\omega_{2}^{2}E_{12}^{\prime }+2\omega_{1}\omega_{2}E_{13}^{\prime }-2\omega_{1}E_{14}^{\prime }-2\omega_{2}E_{15}^{\prime }\right)$$

where

$$v=\frac{m_{1}\mu_{x}}{m_{1}\mu_{x}+m_{2}},k_{a}^{\prime }=\frac{1}{2}\left\{k_{2}^{2}+k_{2}\left(2k_{1}+1\right)+2k_{1}^{2}\right\}$$

$$E_{11}^{\prime }=1+b\left\{\eta_{40}^{*}+3k_{1}^{2}\eta_{04}^{*}+4\eta_{03}C_{x}k_{1}k_{3}v+C_{x}^{2}k_{3}v^{2}\left(2k_{3}+1\right)-4\theta_{a}^{*}k_{1}-4\eta_{21}{C_{x}k}_{3}v\right\}$$

$$E_{12}^{\prime }=1+b\left\{\eta_{40}^{*}+k_{2}\left(2k_{2}+1\right)\eta_{04}^{*}+4k_{2}vk_{3}\eta_{03}C_{x}+C_{x}^{2}k_{3}v^{2}\left(2k_{3}+1\right)-4\theta_{a}^{*}k_{2}-4\eta_{21}{C_{x}k}_{3}v\right\}$$

$$E_{13}^{\prime }=1+b\left\{\eta_{40}^{*}+\eta_{04}^{*}k_{a}^{1}+2\eta_{03}C_{x}k_{3}v\left(k_{2}+k_{1}\right)+C_{x}^{2}k_{3}v^{2}\left(2k_{3}+1\right)-\left(2k_{2}+2k_{1}\right)\theta_{a}^{*}-4\eta_{21}{C_{x}k}_{3}v\right\}$$

$$E_{14}^{\prime }=1+b\left\{k_{1}^{2}\eta_{04}^{*}-\eta_{03}C_{x}{k_{1}k}_{3}v+\frac{C_{x}^{2}}{2}k_{3}v^{2}\left(k_{3}+1\right)-\left(k_{1}\theta_{a}^{*}+vk_{3}\eta_{21}C_{x}\right)\right\}$$

and

$$E_{15}^{\prime }=1+b\left\{\frac{1}{2}\eta_{04}^{*}k_{2}\left(k_{2}+1\right)+k_{2}vk_{3}\sqrt{\eta_{03}}C_{x}+\frac{1}{2}C_{x}^{2}k_{3}v^{2}\left(k_{3}+1\right)-\theta_{a}^{*}k_{2}-\eta_{21}{C_{x}k}_{3}v\right\}$$

The MSE of $F_{g}^{\prime }$ is optimum if

$$\omega_{1}=\frac{E_{12}^{\prime }E_{14}^{\prime }-E_{13}^{\prime }E_{15}^{\prime }}{E_{11}^{\prime }E_{12}^{\prime }-E_{13}^{\prime 2}}=\omega_{1}^{\left(o\right)}\;\left(say\right)\text{,}$$

$$\omega_{2}=\frac{E_{12}^{\prime }E_{15}^{\prime }-E_{14}^{\prime }E_{13}^{\prime }}{E_{11}^{\prime }E_{12}^{\prime }-E_{13}^{\prime 2}}=\omega_{2}^{\left(o\right)}\;\left(say\right)\text{,}$$

(17) $$MSE{\left(F_{g}^{\prime }\right)}_{\min}=\mu_{40}\left\{1-\frac{E_{12}^{\prime }E_{14}^{\prime 2}-2E_{13}^{\prime }E_{14}^{\prime }E_{15}^{\prime }+E_{11}^{\prime }E_{15}^{\prime 2}}{E_{11}^{\prime }E_{12}^{\prime }-E_{13}^{\prime 2}}\right\}$$

Some members of $\left(F_{g}^{\prime }\right),g=\left(1,2,\ldots ,16\right)$ are displayed in Table 4.

**Table 4:** Family members of $F_{g}^{\prime }$.

Estimator	Values of constant				
	$m_{1}$	$m_{2}$	$k_{1}$	$k_{2}$	$k_{3}$
$F_{g^{1}\;}^{\prime }=\left\{\omega_{1}{\hat{\mu }}_{20}\left(\frac{\mu_{02}}{{2\mu }_{02}-{\hat{\mu }}_{02}}\right)+\omega_{2}{\hat{\mu }}_{20}\left(\frac{\mu_{02}}{{\hat{\mu }}_{02}}\right)\right\}\left(\frac{H_{Lx}\mu_{x}+M_{Rx}}{H_{Lx}{\hat{\mu }}_{x}+M_{Rx}}\right)$	$H_{Lx}$	$M_{Rx}$	−1	1	1
$F_{g^{2}}^{\prime }=\left\{\omega_{1}{\hat{\mu }}_{20}\left(\frac{\mu_{02}}{{2\mu }_{02}-{\hat{\mu }}_{02}}\right)+\omega_{2}{\hat{\mu }}_{20}\left(\frac{\mu_{02}}{{\hat{\mu }}_{02}}\right)\right\}\left(\frac{{M_{Rx}\mu }_{x}+D_{Mx}}{M_{Rx}{\hat{\mu }}_{x}+D_{Mx}}\right)$	$M_{Rx}$	$D_{Mx}$	−1	1	1
$F_{g^{3}\;}^{\prime }=\left\{\omega_{1}{\hat{\mu }}_{20}\left(\frac{\mu_{02}}{{2\mu }_{02}-{\hat{\mu }}_{02}}\right)+\omega_{2}{\hat{\mu }}_{20}\left(\frac{\mu_{02}}{{\hat{\mu }}_{02}}\right)\right\}\left(\frac{H_{Lx}\mu_{x}+Q_{rx}}{H_{Lx}{\hat{\mu }}_{x}+Q_{rx}}\right)$	$H_{Lx}$	$Q_{rx}$	−1	1	1
$F_{g^{4}\;}^{\prime }=\left\{\omega_{1}{\hat{\mu }}_{20}\left(\frac{\mu_{02}}{{2\mu }_{02}-{\hat{\mu }}_{02}}\right)+\omega_{2}{\hat{\mu }}_{20}\left(\frac{\mu_{02}}{{\hat{\mu }}_{02}}\right)\right\}\left(\frac{D_{Mx}\mu_{x}+\eta_{03}}{D_{Mx}{\hat{\mu }}_{x}+\eta_{03}}\right)$	$D_{Mx}$	$\eta_{03}$	−1	1	1
$F_{g^{5}\;}^{\prime }=\left\{\omega_{1}{\hat{\mu }}_{20}\left(\frac{\mu_{02}}{{2\mu }_{02}-{\hat{\mu }}_{02}}\right)+\omega_{2}{\hat{\mu }}_{20}\right\}{\left(\frac{\eta_{04}^{*}\mu_{x}+C_{x}}{\eta_{04}^{*}{\hat{\mu }}_{x}+C_{x}}\right)}^{1}$	$\eta_{04}^{*}$	$C_{x}$	−1	0	1
$F_{g^{6}}^{\prime }=\left\{\omega_{1}{\hat{\mu }}_{20}\left(\frac{\mu_{02}}{{2\mu }_{02}-{\hat{\mu }}_{02}}\right)+\omega_{2}{\hat{\mu }}_{20}\left(\frac{\mu_{02}}{{\hat{\mu }}_{02}}\right)\right\}\left(\frac{{M_{Rx}\mu }_{x}+D_{10x}}{M_{Rx}{\hat{\mu }}_{x}+D_{10x}}\right)$	$M_{Rx}$	$D_{10x}$	−1	1	1
$F_{g^{7}\;}^{\prime }=\left\{\omega_{1}{\hat{\mu }}_{20}\left(\frac{\mu_{02}}{{2\mu }_{02}-{\hat{\mu }}_{02}}\right)+\omega_{2}{\hat{\mu }}_{20}\right\}{\left(\frac{H_{Lx}\mu_{x}+D_{Mx}}{H_{Lx}{\hat{\mu }}_{x}+D_{Mx}}\right)}^{1}$	$H_{Lx}$	$D_{Mx}$	−1	0	1
$F_{g^{8}\;}^{\prime }=\left\{\omega_{1}{\hat{\mu }}_{20}\left(\frac{\mu_{02}}{{\hat{\mu }}_{02}}\right)+\omega_{2}{\hat{\mu }}_{20}{\left(\frac{\mu_{02}}{{\hat{\mu }}_{02}}\right)}^{-1}\right\}{\left(\frac{Q_{1x}\mu_{x}+\eta_{04}}{Q_{1x}{\hat{\mu }}_{x}+\eta_{04}}\right)}^{-1}$	$Q_{1x}$	$\eta_{04}$	1	−1	−1
$F_{g^{9}}^{\prime }=\left\{\omega_{1}{\hat{\mu }}_{20}\left(\frac{\mu_{02}}{{2\mu }_{02}-{\hat{\mu }}_{02}}\right)+\omega_{2}{\hat{\mu }}_{20}\left(\frac{\mu_{02}}{{\hat{\mu }}_{02}}\right)\right\}\left(\frac{{M_{Tri}\mu }_{x}+C_{x}}{M_{Tri}{\hat{\mu }}_{x}+C_{x}}\right)$	$M_{Tri}$	$C_{x}$	−1	1	1
$F_{g^{10}\;}^{\prime }=\left\{\omega_{1}{\hat{\mu }}_{20}\left(\frac{\mu_{02}}{{\hat{\mu }}_{02}}\right)+\omega_{2}{\hat{\mu }}_{20}{\left(\frac{\mu_{02}}{{\hat{\mu }}_{02}}\right)}^{-1}\right\}{\left(\frac{H_{Lx}\mu_{x}+\eta_{03}}{H_{Lx}{\hat{\mu }}_{x}+\eta_{03}}\right)}^{-1}$	$H_{Lx}$	$\eta_{03}$	1	−1	−1
$F_{g^{11}\;}^{\prime }=\left\{\omega_{1}{\hat{\mu }}_{20}\left(\frac{\mu_{02}}{{2\mu }_{02}-{\hat{\mu }}_{02}}\right)+\omega_{2}{\hat{\mu }}_{20}\left(\frac{\mu_{02}}{{\hat{\mu }}_{02}}\right)\right\}\left(\frac{H_{Lx}\mu_{x}+\eta_{04}}{H_{Lx}{\hat{\mu }}_{x}+\eta_{04}}\right)$	$H_{Lx}$	${\;\eta }_{04}$	−1	1	1
$F_{g^{12}}^{\prime }=\left\{\omega_{1}{\hat{\mu }}_{20}\left(\frac{\mu_{02}}{{2\mu }_{02}-{\hat{\mu }}_{02}}\right)+\omega_{2}{\hat{\mu }}_{20}\left(\frac{\mu_{02}}{{\hat{\mu }}_{02}}\right)\right\}\left(\frac{H_{Lx}\mu_{x}+D_{Mx}}{H_{Lx}{\hat{\mu }}_{x}+D_{Mx}}\right)$	$H_{Lx}$	$D_{Mx}$	−1	1	1
$F_{g^{13}}^{\prime }=\left\{\omega_{1}{\hat{\mu }}_{20}\left(\frac{\mu_{02}}{{2\mu }_{02}-{\hat{\mu }}_{02}}\right)+\omega_{2}{\hat{\mu }}_{20}\left(\frac{\mu_{02}}{{\hat{\mu }}_{02}}\right)\right\}\left(\frac{{M_{Rx}\mu }_{x}+Q_{rx}}{M_{Rx}{\hat{\mu }}_{x}+Q_{rx}}\right)$	$M_{Rx}$	$Q_{rx}$	−1	1	1
$F_{g^{14}\;}^{\prime }=\left\{\omega_{1}{\hat{\mu }}_{20}\left(\frac{\mu_{02}}{{2\mu }_{02}-{\hat{\mu }}_{02}}\right)+\omega_{2}{\hat{\mu }}_{20}\left(\frac{\mu_{02}}{{\hat{\mu }}_{02}}\right)\right\}\left(\frac{H_{Lx}\mu_{x}+Q_{3x}}{H_{Lx}{\hat{\mu }}_{x}+Q_{3x}}\right)$	$H_{Lx}$	$Q_{3x}$	−1	1	1
$F_{g^{15}}^{\prime }=\left\{\omega_{1}{\hat{\mu }}_{20}\left(\frac{\mu_{02}}{{2\mu }_{02}-{\hat{\mu }}_{02}}\right)+\omega_{2}{\hat{\mu }}_{20}\right\}{\left(\frac{\mu_{x}^{2}+C_{x}}{{\hat{\mu }}_{x}\mu_{x}+C_{x}}\right)}^{1}$	$\mu_{x}$	$C_{x}$	−1	0	1
$F_{g^{16}\;}^{\prime }=\left\{\omega_{1}{\hat{\mu }}_{20}\left(\frac{\mu_{02}}{{\hat{\mu }}_{02}}\right)+\omega_{2}{\hat{\mu }}_{20}{\left(\frac{\mu_{02}}{{\hat{\mu }}_{02}}\right)}^{-1}\right\}{\left(\frac{\eta_{04}\mu_{x}+\eta_{03}}{\eta_{04}{\hat{\mu }}_{x}+\eta_{03}}\right)}^{-1}$	${\;\eta }_{04}$	$\eta_{03}$	1	−1	−1

Now, as particular case, taking a constraint ($\omega_{1}$ + $\omega_{2}$ = 1), we obtained equation (18)

(18) $$F_{g}^{\prime \prime }=\left[\omega_{1}{\hat{\mu }}_{20}\left\{\frac{\mu_{02}}{k_{1}{\hat{\mu }}_{02}+\left(1-k_{1}\right)\mu_{02}}\right\}+\left(1-\omega_{1}\right){\hat{\mu }}_{20}{\left(\frac{\mu_{02}}{{\hat{\mu }}_{02}}\right)}^{k_{2}}\right]{\left(\frac{m_{1}\mu_{x}+m_{2}}{m_{1}{\hat{\mu }}_{x}+m_{2}}\right)}^{k_{3}}$$

Putting $\omega_{2}=\left(1-\omega_{1}\right)$ in equation (15), and equation (16), we obtained the bias, MSE and asymptotic MSE of $F_{g}^{\prime \prime }$ in equation (19), equation (20), and equation (21), as:

(19) $$B\left(F_{g}^{\prime \prime }\right)=b\mu_{20}\left(d_{11}+d_{22}-d_{33}-1\right)$$

where

$$d_{11}=\left(\omega_{1}k_{1}^{2}\eta_{04}^{*}-\omega_{1}{k_{1}\theta }_{a}^{*}+\omega_{2}\right)\text{,}$$

$$d_{22}=\left\{\omega_{2}\frac{k_{2}\left(k_{2}+1\right)}{2}\eta_{04}^{*}-\omega_{2}{k_{2}\theta }_{a}^{*}+\omega_{1}v^{2}\frac{k_{3}\left(k_{3}+1\right)}{2}C_{x}^{2}+\omega_{1}k_{1}vk_{3}\sqrt{\eta_{03}\;}C_{x}\right\}\text{,}$$

$$d_{33}=\left\{\omega_{1}vk_{3}\eta_{21}C_{x}+\omega_{2}v^{2}\frac{k_{3}\left(k_{3}+1\right)}{2}C_{x}^{2}+\omega_{2}{k_{2}vk_{3}\theta }_{a\;}^{*}-\omega_{2}vk_{3}\eta_{21}C_{x}\right\}\text{.}$$

and

(20) $$MSE\left(F_{g}^{\prime \prime }\right)=\mu_{40}\left\{1+E_{12}^{\prime }-2E_{15}^{\prime }+\omega_{1}^{2}\left(E_{11}^{\prime }+E_{12}^{\prime }-{2E}_{13}^{\prime }\right)-2\omega_{1}\left(E_{12}^{\prime }-E_{13}^{\prime }+E_{14}^{\prime }-E_{15}^{\prime }\right)\right\}$$

The MSE of $F_{g}^{\prime \prime }$ is optimum when

$$\omega_{1}=\frac{E_{12}^{\prime }-E_{13}^{\prime }+E_{14}^{\prime }-E_{15}^{\prime }}{E_{11}^{\prime }+E_{12}^{\prime }-{2E}_{13}^{\prime }}=\omega_{1}^{\left(0\right)}\;\left(\text{say}\right)$$

(21) $$MSE{\left(F_{g}^{\prime \prime }\right)}_{\min}=\mu_{40}\left\{1+E_{12}^{\prime }-2E_{15}^{\prime }-\frac{{\left(E_{12}^{\prime }-E_{13}^{\prime }+E_{14}^{\prime }-E_{15}^{\prime }\right)}^{2}}{E_{11}^{\prime }+E_{12}^{\prime }-{2E}_{13}^{\prime }}\right\}$$

## 4 Efficiency comparisons

To compare the suggested class of estimators $F_{g}^{\prime }$ with a usual estimation methods, regression, ratio-type, and different type of estimators, we write the MSEs of all existing estimators, and the minimum MSE of $F_{g}^{\prime }$ to the first degree of approximation (ignoring finite population correction (fpc) term) respectively as:

$$MSE{\left(F_{g}^{\prime \prime }\right)}_{\min}<V\left({\hat{g}}_{0}\right)$$

$$\mu_{40}\left\{1-\frac{\left(E_{12}^{\prime }E_{14}^{\prime 2}-2E_{13}^{\prime }E_{14}^{\prime }E_{15}^{\prime }+E_{11}^{\prime }E_{15}^{\prime 2}\right)}{E_{11}^{\prime }+E_{12}^{\prime }-E_{13}^{\prime 2}}\right\}<b\mu_{40}\eta_{40}^{*}$$

$$\mu_{40}\left\{1-\frac{{\left(E_{12}^{\prime }E_{14}^{\prime 2}-2E_{13}^{\prime }E_{14}^{\prime }E_{15}^{\prime }+E_{11}^{\prime }E_{15}^{\prime 2}\right)}^{2}}{E_{11}^{\prime }+E_{12}^{\prime }-E_{13}^{\prime 2}}\right\}<\mu_{40}\left[1-\frac{M_{a}^{2}}{M_{b}}\right]\;\left(j=1,2\ldots \ldots .16\right)$$

$$\mu_{40}\left\{1-\frac{{\left(E_{12}^{\prime }E_{14}^{\prime 2}-2E_{13}^{\prime }E_{14}^{\prime }E_{15}^{\prime }+E_{11}^{\prime }E_{15}^{\prime 2}\right)}^{2}}{E_{11}^{\prime }+E_{12}^{\prime }-E_{13}^{\prime 2}}\right\}<\mu_{40}\left[1-\frac{M_{c}^{2}}{M_{d}}\right]\;\left(j=1,2\ldots \ldots .16\right)$$

$$\mu_{40}\left\{1-\frac{{\left(E_{12}^{\prime }E_{14}^{\prime 2}-2E_{13}^{\prime }E_{14}^{\prime }E_{15}^{\prime }+E_{11}^{\prime }E_{15}^{\prime 2}\right)}^{2}}{E_{11}^{\prime }+E_{12}^{\prime }-E_{13}^{\prime 2}}\right\}<\left[\mu_{40}-\frac{M_{e}^{2}}{M_{f}}\right]\;\left(j=1,2\ldots \ldots .16\right)$$

From the theoretical results, it is well-established that the proposed class of the estimation methods have better efficiency under the above mentioned certain circumstances.

## 5 Applications

We consider three natural populations for efficiency comparison. The descriptive statistics of populations are given in Table 5, for this we use the same data used by:

Population-I (Pop-I): Taken from Ref. [16] page 177. the description of the study variable Y, auxiliary variable X are shown as:

Y: shows the area of wheat in 1974.
X: shows the area of wheat in 1971.

Population II (Pop-II): Also taken from [16] page 177. These two considered variables are:

Y: is the area under wheat in 1974.
X: is the area under wheat in 1973.

Population III (Pop-III): The data set is taken from Ref. [16] page 177. The characteristics of all the three populations are provided in Table 5.

$$REF=\frac{V\left({\hat{g}}_{0}\right)}{MSE{\left(F_{g}^{\prime }\;or\;T_{Aj},T_{Bj},T_{Cj}\right)}_{\min}}\times 100$$

**Table 5:** Descriptive statistics for different natural populations.

Notation	Pop-I	Pop-II	Pop-III	Notation	Pop-I	Pop-II	Pop-III
N	34	34	34	$Q_{dx}$	8.03	8.94	8.03
n	20	20	20	$Q_{ax}$	17.45	18.86	17.45
$\mu_{y}$	85.64	85.64	129.76	$D_{1x}$	7.03	6.06	7.03
$\mu_{x}$	20.89	19.94	20.89	$D_{2x}$	7.68	8.30	7.68
θa	1.15	1.22	0.78	$D_{3x}$	10.82	10.27	10.82
$C_{y}$	0.86	0.86	0.93	$D_{4x}$	12.94	11.12	12.94
$C_{x}$	0.72	0.75	0.72	$D_{5x}$	15.00	14.25	15.00
$\eta_{04}$	2.91	3.73	2.91	$D_{6x}$	22.72	21.02	22.72
$\eta_{40}$	13.37	13.37	3.54	$D_{7x}$	25.04	26.45	25.04
$\mu_{20}$	73.31	73.31	120.76	$D_{8x}$	33.56	30.44	33.56
$\mu_{02}$	15.05	15.02	15.05	$D_{9x}$	43.61	37.32	43.61
$\eta_{03}$	0.87	1.28	0.87	$D_{10x}$	56.40	63.40	56.40
$\eta_{21}$	−0.31	−0.29	−0.43	$M_{Tri}$	16.23	16.56	16.23
$M_{d}$	15.00	14.25	15.00	$M_{Rx}$	28.45	32.00	28.45
$Q_{1}$	9.43	9.93	9.43	$H_{Lx}$	18.95	18.25	18.95
$Q_{3}$	25.48	27.80	25.48	$D_{Mx}$	19.82	18.36	19.82
$Q_{rx}$	16.05	17.88	16.05	ρ	0.49	0.50	0.31

Efficiency results are provided in Table 6, Table 7, Table 8, Table 9.

**Table 6:** PRE of existing class I estimator of $T_{Aj}$.

Estimators	Pop-I	Pop-II	Pop-III
$T_{A1}$	139.59	140.83	140.78
$T_{A2}$	140.26	141.30	141.25
$T_{A3}$	139.78	140.98	140.93
$T_{A4}$	139.84	141.02	140.97
$T_{A5}$	139.83	141.02	140.96
$T_{A6}$	140.46	141.40	141.36
$T_{A7}$	140.01	141.15	141.10
$T_{A8}$	140.07	141.18	141.13
$T_{A9}$	140.37	141.36	141.31
$T_{A10}$	140.88	141.59	141.55
$T_{A11}$	140.53	141.44	141.39
$T_{A12}$	140.57	141.46	141.41
$T_{A13}$	138.17	139.22	139.14
$T_{A14}$	138.78	140.03	139.96
$T_{A15}$	138.32	139.44	139.37
$T_{A16}$	138.37	139.51	139.43

**Table 7:** PRE of existing class I estimator of $T_{Bj}$.

Estimators	Pop-I	Pop-II	Pop-III
$T_{B1}$	145.80	147.19	147.13
$T_{B2}$	146.69	147.91	147.86
$T_{B3}$	145.93	147.32	147.26
$T_{B4}$	145.94	147.33	147.27
$T_{B5}$	146.52	147.79	147.74
$T_{B6}$	147.30	148.24	148.20
$T_{B7}$	146.65	147.88	147.83
$T_{B8}$	146.65	147.89	147.83
$T_{B9}$	147.16	148.17	148.13
$T_{B10}$	147.76	148.42	148.38
$T_{B11}$	147.26	148.22	148.18
$T_{B12}$	147.27	148.23	148.18
$T_{B13}$	144.51	145.57	145.48
$T_{B14}$	145.25	146.59	146.52
$T_{B15}$	144.60	145.71	145.63
$T_{B16}$	144.61	145.71	145.64

**Table 8:** PRE of existing class II estimator of $T_{Cj\;}$.

Estimators	Pop-I	Pop-II	Pop-III
$T_{C1}$	172.96	187.08	186.97
$T_{C2}$	180.71	194.20	194.09
$T_{C3}$	175.17	189.36	189.24
$T_{C4}$	175.82	189.98	189.87
$T_{C5}$	177.65	191.67	191.56
$T_{C6}$	185.08	197.23	197.13
$T_{C7}$	179.85	193.53	193.42
$T_{C8}$	180.48	194.02	193.92
$T_{C9}$	194.36	201.53	201.44
$T_{C10}$	197.71	202.40	202.31
$T_{C11}$	195.47	201.86	201.77
$T_{C12}$	195.77	201.94	201.85
$T_{C13}$	159.99	169.68	169.55
$T_{C14}$	165.92	178.52	178.40
$T_{C15}$	161.51	172.09	171.97
$T_{C16}$	161.98	172.81	172.69

**Table 9:** PRE of proposed estimator of $F_{g}^{\prime }$.

Estimators	Pop-I	Pop-II	Pop-III
$F_{g^{1}\;}^{\prime }$	213.6412	253.3058	381.5182
$F_{g^{2}\;}^{\prime }$	215.4034	259.3495	414.7740
$F_{g^{3}\;}^{\prime }$	215.0596	257.1394	407.8885
$F_{g^{4}\;}^{\prime }$	216.9833	262.3108	449.2634
$F_{g^{5}\;}^{\prime }$	**232.4338**	**311.5485**	475.7372
$F_{g^{6}\;}^{\prime }$	212.6735	252.2618	365.2024
$F_{g^{7}\;}^{\prime }$	**229.9279**	**302.6034**	431.2467
$F_{g^{8}\;}^{\prime }$	212.1071	256.116	**659.0983**
$F_{g^{9}\;}^{\prime }$	**216.9822**	**262.4609**	449.2356
$F_{g^{10}\;}^{\prime }$	212.7627	258.1215	**703.0769**
$F_{g^{11}\;}^{\prime }$	216.7076	261.4945	442.8807
$F_{g^{12}\;}^{\prime }$	214.6151	257.0012	399.2852
$F_{g^{13}\;}^{\prime }$	215.7126	259.434	421.1429
$F_{g^{14}\;}^{\prime }$	213.9697	254.398	387.3530
$F_{g^{15}\;}^{\prime }$	**234.6482**	**316.1433**	**522.5838**
$F_{g^{16}\;}^{\prime }$	212.1307	256.3242	**660.5909**

## 6 Conclusions

In this study, we have suggested a new class of estimators for variance estimation utilizing some known traditional and robust measures of supplementary information. Using three natural populations, we have computed the PRE of all existing estimators and the findings are summarized in Table 6, Table 7, Table 8, Table 9. It is observed from Table 6, Table 7, Table 8, Table 9 that all members of the proposed class $F_{g}^{\prime }$ (*g* = 1,2, …,16) are superior than the all-existing classes of estimators. It is also examined that our suggested class of estimators is better than all the reviewed estimators. Further, it is concluded that the estimator $F_{g}^{\prime }$ bold values indicate maximum PREs among all the existing estimators. Theoretically and empirically findings of this article are quite instructive. Therefore, the proposed class of estimators $F_{g}^{\prime }$ is confirmed. In future studies, the work can be extended in light of adaptive cluster sampling.

## Data availability statement

Data will be made available on request.

## Funding

This work was supported by the Deanship of Scientific Research, Vice Presidency for Graduate Studies and Scientific Research, 10.13039/501100020912King Faisal University, Saudi Arabia [Grant No. 6058].

## CRediT authorship contribution statement

**Mohammed Ahmed Alomair:** Writing – review & editing, Writing – original draft, Funding acquisition, Formal analysis, Data curation, Conceptualization. **Syed Aflake Hussain Shah Gardazi:** Writing – review & editing, Writing – original draft, Visualization, Validation, Supervision, Software, Resources, Project administration, Methodology, Investigation, Formal analysis, Data curation, Conceptualization.

## Declaration of competing interest

The authors declare that they have no known competing financial interests or personal relationships that could have appeared to influence the work reported in this paper.
